# Supplementary material for: Deletion of wfs1 Impairs Oligodendrocyte Precursor Cells Dorsal Distribution and Myelination Through the wfs1-hmgcs1 Axis in Zebrafish
Source: Biology (Basel). 2026 Aug 16;15(16):1402. doi: 10.3390/biology15161402 (PMC13510151; doi:10.3390/biology15161402)
Supplement: Supplementary file 1 [file biology-15-01402-s001.zip › File S1-Transcriptome sequencing data and differentially expressed genes (DEGs) in wfs1-- zebrafish at 5 dpf/2.KEGG/wfs1KOvsWT/UP/src/dre04012.html]

dre04012


- dre:100149258

- Up regulated genes

544664\_1(3.28411269336583)

- dre:100149258

- Up regulated genes

544664\_1(3.28411269336583)

- dre:556277

- Up regulated genes

556277(2.56614816386233)

- dre:100149258

- Up regulated genes

544664\_1(3.28411269336583)

- dre:100006675

- Up regulated genes

100006675(1.63301233543285)

- dre:100000252

- Up regulated genes

100000252\_3(1.94434154854557) 100000252(1.09163460881879)

Close
